# Effect of day 5 or 6 blastocyst embryo transfer on pregnancy outcomes in euthyroid women undergoing IVF: A single centre retrospective cohort

**DOI:** 10.5935/1518-0557.20240036

**Published:** 2024

**Authors:** Ricardo Andre Medeiros Negreiros, Viviane Rosado Negreiros d’Assunção, Luis Eduardo Negreiros d’Assunção, Maria Madalena Pessoa Caldas, Eduardo Sérgio Soares Sousa

**Affiliations:** 1 Centre for Medical Sciences, Federal University of Paraíba, Paraíba, Brazil; 2 University Center of João Pessoa (UNIPE), Paraíba, Brazil; 3 Geare Centro de Reprodução Humana e Genética, Pernambuco, Brazil

**Keywords:** *in vitro* fertilization, blastocyst, embryo transfer, thyroid peroxidase antibodies, pregnancy outcome

## Abstract

**Objective:**

This study examined whether blastocysts transferred on day 5 or day 6 of embryo development, as well as positivity for anti-thyroid peroxidase antibodies, affect gestational outcomes in euthyroid women undergoing in vitro fertilisation.

**Methods:**

Of 428 women who underwent in vitro fertilisation assessed in this retrospective cohort study, 212 (49.5%) underwent embryo transfer on day 5 of blastulation and 216 (50.5%) on day 6. Dichotomization based on anti-thyroid peroxidase antibodies status was also performed, with 370 (86.4%) women testing negative and 58 (13.6%) testing positive. Clinical and hormonal data and rates of clinical pregnancy, miscarriage, and live births were compared between the groups.

**Results:**

When evaluating gestational outcomes based on the day of blastulation, a statistically significant difference was observed in clinical pregnancy rates [51.4% (day 5) *vs*. 40.7% (day 6); *p*=0.033]. However, there was no significant difference in the relative frequencies of miscarriages (*p*=1.000), live births (*p*=1.000), or preterm births (*p=*1.000). Using Cramer’s V test, a weak association was found between the day of blastulation and clinical pregnancy outcomes (V^2^=10.7%; *p*=0.027). There were no statistically significant differences between the anti-thyroid peroxidase antibodies-negative and -positive groups in terms of clinical pregnancy rates (*p*=0.396), miscarriages (*p*=0.129), and live births (*p*=0.129).

**Conclusions:**

Higher rates of clinical pregnancy were observed in women who underwent embryo transfers performed on day 5 compared to those on day 6. However, no effect was observed with gestational outcomes. Further, anti-thyroid peroxidase antibody positivity did not have a statistically significant impact on gestational outcomes.

## INTRODUCTION

The use of assisted reproductive techniques (ART) has increased globally owing to technological advancements in diagnostic and infertility treatment methods (Kushnir *et al*., 2017). For instance, in vitro fertilisation (IVF) techniques have advanced significantly in the field of embryo culture, culminating in the maturation of embryos to the blastocyst stage and detailed morphological evaluation for improved embryo selection (Eskew & Jungheim, 2017; Lundin & Park, 2020). However, ART still has limitations, such as lower therapeutic efficacy rates and live birth rates than the rates of unsuccessful cycles. In addition, there are stressful and financial components involved in the procedures. It is necessary to evaluate the individual predictive factors that favour a better treatment prognosis (Kushnir *et al*., 2017; Szamatowicz, 2016).

The elapsed time of embryonic maturation after IVF appears to play a crucial role in gestational outcomes, suggesting potential advantages of embryo transfer at the blastocyst stage (5-6 days after fertilisation) compared to the cleavage stage (2-3 days after fertilisation). This is because the former favours the selection of embryos with a higher implantation potential, and the timing of embryo exposure to the uterus is closer to the natural menstrual cycle (Martins *et al*., 2017).

Despite inconclusive findings when evaluating gestational outcomes, studies have suggested the possibility of higher rates of clinical pregnancy and live births in embryos transferred at the blastocyst stage compared to the cleavage stage (Glujovsky *et al*., 2022), while others have found no statistical significance between the two stages (Garbhini *et al*., 2023; Günther *et al*., 2022; Martins *et al*., 2017). Some centres have opted for blastocyst stage embryo transfers after a failure with cleavage stage embryos (Barrenetxea *et al*., 2005; Orvieto *et al*., 2022).

When evaluating differences between blastocyst-stage embryos transferred on Day 5 (D5) or Day 6 (D6) of development, published findings indicate a higher occurrence of favourable gestational outcomes, such as a significantly higher probability of implantation, clinical pregnancy, and live births for embryos transferred on D5 than for those transferred on D6 (Coticchio *et al*., 2023; Li *et al*., 2020). However, other studies have found no significance in some of these parameters (Andrabi *et al*., 2022). Factors such as chromosomal status (euploidy or not), timing of embryonic expansion, and the number of embryos transferred may be related to the observed effects, suggesting the need for further studies to confirm these results (Elgindy & Elsedeek, 2012; Li *et al*., 2020; Tong *et al*., 2022).

Embryo quality also appears to be a significant prognostic factor, as no significant differences in gestational outcomes were observed when comparing low-quality embryos on D5 with high-quality embryos on D6 (Zhang *et al*., 2023), or when both groups had high-quality embryos (Yang *et al*., 2016). On the other hand, several factors can extend embryonic progression to D6, such as a younger age of the egg provider, the presence of an early blastocyst on D5, and cycles involving surgically retrieved spermatozoa (Wu *et al*., 2023). Therefore, the practice of frozen embryo transfer (FET) cycles with D6 embryos remains a reality. Hence, it is imperative to continue investigating the influence of the timing of embryo transfer and its consequences.

Beyond embryonic factors, there are other variables that might be related to the chances of pregnancy after IVF, such as age, duration of infertility/time of conception, number of retrieved oocytes, and metabolic/endocrine factors, such as basal follicle-stimulating hormone (FSH) levels and thyroid function (d’Assunção *et al*., 2022; van Loendersloot *et al*., 2010).

Thyroid autoimmunity, assessed by the presence of circulating anti-thyroid peroxidase antibodies (TPOAb), can alter endocrine function, and therefore, may adversely affect reproductive outcomes in pregnancies induced by ART (Bucci *et al*., 2022; Dhillon-Smith & Coomarasamy, 2020; Xu *et al*., 2023).

The presence of TPOAb has been noted in several studies as a condition associated with recurrent miscarriages, and supplementation with levothyroxine is recommended for women positive for antibodies, even if euthyroid (Ghalib *et al*., 2023; Poppe *et al*., 2021; Xie *et al*., 2020), although controversial (Dhillon-Smith *et al*., 2019; Wang *et al*., 2020).

Here, a retrospective cohort study was conducted to investigate whether embryo transfer on blastulation D5 or D6 affected the rates of clinical pregnancy, miscarriage, live births, and prematurity in euthyroid women undergoing IVF/intracytoplasmic sperm injection (IVF/ICSI). We also aimed to evaluate the effects of TPOAb on gestational outcomes. This is the first Brazilian study to evaluate such themes.

## MATERIAL AND METHODS

This retrospective cohort study analysed the data of patients who underwent IVF/ICSI treatment followed by blastocyst-stage FET between January 2010 and December 2017 at the Geare Centre for Reproductive Medicine in Recife, Brazil. The study was conducted following the guidelines outlined in the Strengthening the Reporting of Observational Studies in Epidemiology (STROBE) statement (von Elm *et al*., 2007) after approval from the Research Ethics Committee of the Centre for Medical Sciences of the Federal University of Paraíba (approval code CAAE 25654719.5.0000.8069). As this study was retrospective, involved no experimental intervention, and utilised anonymous analysis of data collected from the participants’ medical records, the ethics and research committee waived the requirement for obtaining informed consent from the participants.

### Patients and population

Clinical data and gestational outcomes were collected from women who underwent IVF/ICSI with frozen embryos transferred at the blastocyst stage. The study included women aged ≤45years, with free thyroxine (FT4) levels between ≥0.7ng/dL and ≤1.8ng/dL, and thyroid-stimulating hormone (TSH) levels between ≥0.5mIU/mL and ≤4.5mIU/mL. Women with polycystic ovary syndrome, a history of ovarian or uterine surgery, current or past neoplastic conditions, current or past supplementation with levothyroxine, and those who did not undergo embryo transfer on D5 or D6 of blastulation were excluded. Of the initial 627 patients who met the inclusion criteria, 428 were included in the study after applying the exclusion criteria.

The parameters collected and evaluated in this study included age, body mass index (BMI), time to conceive (in months), FSH and luteinizing hormone (LH) serum concentrations measured in the follicular phase of the menstrual cycle, oestradiol, prolactin, TSH, FT4, TPOAb, retrieved oocytes, metaphase II oocytes (MII), generated embryos, and number of transferred embryos.

For the main analysis, patients were categorised into two groups for comparison based on either D5 (n=212; 49.5%) or D6 (n=216; 50.5%) blastulation during embryo transfer. Dichotomization was also performed based on TPOAb status: negative (n=370; 86.4%) and positive (n=58; 13.6%). In subsequent investigations, differences in the clinical data and hormonal profiles of the patients were evaluated based on clinical pregnancy outcomes: no (n=231; 54%) and yes (n=197; 46%). In each analysis, the clinical and hormonal data, as well as the rates of clinical pregnancy, miscarriage, and live births were compared between the groups.

Clinical pregnancy outcomes were assessed based on the presence of a gestational sac and foetal heartbeat, which were verified using transvaginal ultrasonography performed 4 weeks after embryo transfer.

### Ovarian stimulation, embryo development, and embryo transfer

Patients underwent controlled ovarian stimulation on the 1st or 3rd day of their menstrual cycle using a formulation of recombinant LH and FSH, the latter being either follitropin alpha (Gonal F, Serono, Switzerland) or follitropin beta (Pergoveris, Merck, Germany), for an average duration of 10 days. Doses were adjusted based on the ovarian response determined by transvaginal ultrasound. All patients used gonadotropin hormone-releasing hormone antagonist protocols (Cetrotide; Merck, Germany) when at least one follicle measuring at least 14mm was identified after the 6th day of controlled stimulation with simultaneous administration of gonadotropins. When at least three follicles measuring between 17-22mm were identified, gonadotropin hormone-releasing hormone analogues (Lupron, Abbott, USA) were administered to induce oocyte maturation. Oocyte retrieval was performed through ovarian puncture 36h later, with the IVF/ICSI procedure performed 4 h after retrieval.

Fertilised embryos were cultured until they reached the blastocyst stage and maintained until the 5th or 6th day of maturation. Subsequently, all the samples were subjected to biopsy and frozen by vitrification using the Cryotop method (Kitazato, Japan) (Kuwayama *et al*., 2005). The patient was administered oestradiol valerate (6mg/day) (Primogyna, Bayer, Germany) between the 1st and 3rd day of the menstrual cycle for 10-12 days. Subsequently, the patient was re-evaluated using transvaginal ultrasonography. When the patient’s endometrial thickness was >7mm, serum oestradiol levels were 200-300pg/mL, and progesterone was <1ng/mL, vaginal progesterone (800mg/day) (Utrogestan, Besins Health Care, France) was administered for 5 days for all patients, regardless of the intended embryo blastulation day. After this process, up to two thawed euploid embryos were transferred.

### Statistical analysis

Statistical analysis was conducted using Statistical Package for the Social Sciences statistical software version 26 (SPSS Inc., Chicago, IL, USA). Normality of the data was assessed using the Shapiro-Wilk test. Numerical descriptive data are presented as median and interquartile interval (IQR), and qualitative data are presented as absolute and relative frequencies. The Mann-Whitney U test and Fisher’s exact test were used to assess statistical differences between groups. Cramer’s V test was used to assess the strength of the association between the results and statistical significance. Statistical significance was set at *p*<0.05.

## RESULTS

### Baseline characteristics

Table 1 shows the clinical data, hormonal profiles, ovarian collection, and reproductive outcomes of all participants included in this study.

**Table 1:** Clinical data, hormonal profile and reproductive outcomes of infertile euthyroid women participating in this study.

Characteristics^*^	Median [IQR]
	(*n=*428)
Female age (years)	35 [32 - 38]
BMI (kg/cm^2^)	22.96 [21.36 - 25.02]
Time trying to conceive (months)	24 [12 - 42]
Baseline FSH (UI/mL)	6.56 [5.30 - 7.73]
LH (mUI/mL)	5.50 [4.00 - 7.11]
Estradiol (pg/dL)	47.77 [33.00 - 71.37]
Prolactin (ng/mL)	13.02 [9.20 - 17.83]
TSH (mUI/mL)	1.76 [1.17 - 2.37]
FT4 (ng/dL)	1.14 [1.00 - 1.26]
Thyroid Peroxidase Antibody positive (*n*, %)	58 (13.6%)
Oocytes retrieved (*n*)	9 [5 - 14]
MII oocytes (*n*)	7 [4 - 11]
Embryos (*n*)	4 [2 - 6]
Embryos transferred (*n*)	2 [1 - 2]
Clinical Pregnancy rate (*n*, %)	197 (46%)
Miscarriage (*n*, %)	23 (11.7%)
Live births (*n*, %)	174 (88.3%)
Twins (*n*, %)	44 (25.3%)
Preterm (*n*, %)	31 (17.8%)

* Qualitative variables are presented as absolute and relative frequencies, and quantitative variables are presented as mean and standard deviation and/or median and interquartile range (IQR). BMI, Body Mass Index; FSH: Follicle-stimulating hormone; LH: Luteinizing hormone; TSH, thyroid-stimulating hormone; FT4, free Thyroxine MII: Metaphase II oocytes.

### Embryo culture Day 5 and Day 6

**Figure 1:**
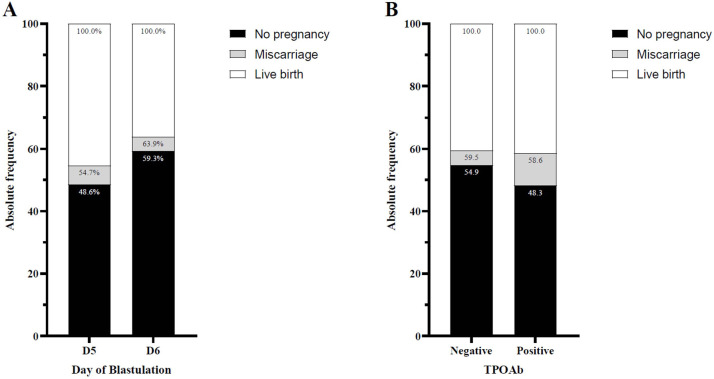
Absolute frequency of reproductive outcomes in infertile euthyroid women according to (A) blastocyst embryo transfer on day five (D5) or day six (D6) and (B) negative or positive anti-thyroxidase antibodies (TPOAb).

The clinical data and gestational outcomes based on the embryo culture time for either D5 or D6 for transfer are presented in Table 2 and schematically depicted in Figure 1A. Statistically significant differences were observed between the groups with respect to age (*p*<0.001), TSH levels (*p*=0.023), number of transferred embryos (*p*<0.001), and clinical pregnancy rates (*p*=0.033). Notably, the groups did not differ in terms of TPOAb positivity (*p*=0.778).

**Table 2:** Clinical data, hormonal profiles, and reproductive outcomes of euthyroid-infertile women according to blastocyst embryo transfer on day five (D5) or day six (D6).

Characteristics^*^	Day of Blastulation		*p*-value
	D5	D6	
*n*	212 (49.5%)	216 (50.5%)	-
Female age (years)	34 [32 - 36]	36 [33 - 40]	**<0.001ª**
BMI (kg/cm^2^)	22.83 [21.36 - 25.00]	23.13 [21.33 - 25.22]	0.738ª
Time trying to conceive (months)	24 [14.25 - 48]	24 [12 - 36]	0.357ª
Baseline FSH (UI/mL)	6.56 [5.32 - 7.80]	6.58 [5.24 - 7.69]	0.851ª
LH (mUI/mL)	5.59 [4.07 - 7.36]	5.34 [3.87 - 6.97]	0.279ª
Estradiol (pg/dL)	47.70 [32.80 - 70.87]	49.00 [33.92 - 73.36]	0.446ª
Prolactin (ng/mL)	13.22 [9.10 - 17.68]	13.00 [9.23 - 17.88]	0.957ª
TSH (mUI/mL)	1.66 [1.10 - 2.30]	1.9 [1.32 - 2.45]	**0.023ª**
FT4 (ng/dL)	1.15 [1.02 - 1.26]	1.13 [0.99 - 1.26]	0.299ª
Thyroid Peroxidase Antibody positive (*n*, %)	30 (14.2%)	28 (13.0%)	0.778^b^
Oocytes retrieved (*n*)	8 [5 - 14]	9 [5 - 13]	0.783ª
MII oocytes (*n*)	6 [4 - 11]	7.5 [4 - 11]	0.801ª
Embryos (*n*)	4 [2 - 6]	4 [2 - 6]	0.976ª
Embryos transferred (*n*)	2 [1 - 2]	2 [1 - 2]	**<0.001**ª
Clinical Pregnancy rate (*n*, %)	109 (51.4%)	88 (40.7%)	**0.033^b^**
Miscarriage (*n*, %)	13 (11.9%)	10 (11.4%)	1.000^b^
Live births (*n*, %)	96 (88.1%)	78 (88.6%)	1.000^b^
Twins (*n*, %)	26 (27.1%)	18 (23.1%)	0.601^b^
Preterm (*n*, %)	17 (17.7%)	14 (17.9%)	1.000^b^

* Qualitative variables are presented as absolute and relative frequencies and quantitative variables as median and interquartile interval (IQR). BMI, Body Mass Index; FSH: Follicle-stimulating hormone; LH: Luteinizing hormone; TSH, thyroid-stimulating hormone; FT4, free Thyroxine MII: Metaphase II oocytes. ª: Mann-Whitney U test.

b Fisher’s Exact test.

When using Cramer’s V test, weak statistically significant associations were found between blastulation day and the number of transferred embryos (V^2^=18%; *p*<0.001) or clinical pregnancy (V^2^=10.7%; *p*=0.027). There were no statistically significant differences in the evaluation of miscarriage and live birth rates relative to clinical pregnancy rates.

### TPOAb pregnancy outcomes

Table 3 shows the gestational outcome data based on TPOAb positivity or negativity, as shown in Figure 1B. Statistically significant differences were only observed for LH (*p*=0.004), TSH (*p*=0.006), and number of twins (*p*=0.010). There were no statistically significant differences in clinical pregnancy, miscarriage, or live birth rates, although the percentage values favoured the TPOAb-negative group.

**Table 3:** Clinical data, hormonal profile, and reproductive outcomes of infertile euthyroid women according to negative or positive anti-thyroxidase antibody (TPOAb).

Characteristics^*^	TPOAb		*p*-value
	Negative	Positive	
*n*	370 (86.4%)	58 (13.6%)	-
Female age (years)	35 [32 - 38]	35 [32 - 37]	0.602ª
BMI (kg/cm^2^)	23.11 [21.30 - 25.03]	22.83 [21.64 - 25.09]	0.883ª
Time trying to conceive (months)	24 [12 - 42]	24 [16.5 - 48]	0.740ª
Baseline FSH (UI/mL)	6.6 [5.32 - 7.73]	6.3 [4.98 - 8.45]	0.551ª
LH (mUI/mL)	5.3 [3.90 - 6.95]	6.2 [4.57 - 8.07]	**0.004ª**
Estradiol (pg/dL)	48.15 [33.7 - 74.02]	41.95 [27 - 55]	0.019ª
Prolactin (ng/mL)	13.4 [9.10 - 17.85]	12.65 [9.75 - 17.49]	0.986ª
TSH (mUI/mL)	1.7 [1.13 - 2.31]	2.29 [1.46 - 3.07]	**0.006ª**
FT4 (ng/dL)	1.13 [1.00 - 1.26]	1.17 [1.00 - 1.30]	0.162ª
Oocytes retrieved (*n*)	9 [6 - 15]	13 [8 - 17.75]	0.443ª
MII oocytes (*n*)	7 [5 - 12]	10.5 [6 - 14.75]	0.594ª
Embryos (*n*)	4 [3 - 7]	6 [3.25 - 7.75]	0.369ª
Embryos transferred (*n*)	2 [2 - 2]	2 [1 - 2]	0.291ª
Clinical Pregnancy rate (*n*, %)	167 (45.1%)	30 (51.7%)	0.396^b^
Miscarriage (*n*, %)	17 (10.2%)	6 (20%)	0.129^b^
Live births (*n*, %)	150 (89.8%)	24 (80%)	0.129^b^
Twins (*n*, %)	43 (28.7%)	1 (4.2%)	**0.010^b^**
Preterm (*n*, %)	28 (18.7%)	3 (12.5%)	0.576^b^

* Qualitative variables are presented as absolute and relative frequencies and quantitative variables as median and interquartile interval (IQR). BMI, Body Mass Index; FSH: Follicle-stimulating hormone; LH: Luteinizing hormone; TSH, thyroid-stimulating hormone; FT4, free Thyroxine MII: Metaphase II oocytes. ª: Mann-Whitney U test.

b Fisher’s Exact test.

Cramer’s V test revealed a weak association between twin pregnancies and TPOAb negativity (V^2^=19.4%; *p*=0.010).

### Overall pregnancy outcomes

Table 4 presents the findings according to the clinical pregnancy outcome groups (No or Yes), revealing statistically significant differences in age (*p*=0.007), serum LH levels (*p*=0.011), number of retrieved oocytes (*p*<0.001), number of metaphase II oocytes (*p*<0.001), number of generated embryos (*p*<0.001), and number of transferred embryos (*p*<0.001).

**Table 4:** Clinical data and hormonal profiles of infertile euthyroid women according to clinical pregnancy outcomes.

Characteristics^*^	Clinical Pregnancy		*p*-value
	No	Yes	
n	231 (54%)	197 (46%)	-
Female age (years)	36 [32 - 39]	34 [32 - 37]	**0.007ª**
BMI (kg/cm^2^)	23.23 [21.71 - 25.63]	22.83 [21.22 - 24.64]	0.135ª
Time trying to conceive (months)	24 [12 - 48]	24 [12 - 39]	0.867ª
Baseline FSH (UI/mL)	6.47 [5.31 - 7.70]	6.60 [5.30 - 7.91]	0.659ª
LH (mUI/mL)	5.20 [3.79 - 6.70]	5.91 [4.39 - 7.55]	**0.011ª**
Estradiol (pg/dL)	48.30 [34.00 - 72.00]	46.63 [32.05 - 71.05]	0.438ª
Prolactin (ng/mL)	13.00 [9.30 - 17.90]	13.40 [9.17 - 17.48]	0.684ª
TSH (mUI/mL)	1.80 [1.13 - 2.46]	1.71 [1.23 - 2.35]	0.783ª
FT4 (ng/dL)	1.14 [1.00 - 1.29]	1.14 [1.02 - 1.25]	0.534ª
Thyroid Peroxidase Antibody positive (n, %)	28 (12.1%)	30 (15.2%)	0.396^b^
Oocytes retrieved (n)	8 [4 - 12]	10 [6 - 15]	**<0.001^b^**
MII oocytes (n)	6 [3 - 10]	8 [5 - 12.5]	**<0.001^b^**
Embryos (n)	3 [2 - 5]	5 [3 - 7]	**<0.001^b^**
Embryos transferred (n)	2 [1 - 2]	2 [1 - 2]	**<0.001^b^**

* Qualitative variables are presented as absolute and relative frequencies and quantitative variables as median and interquartile interval (IQR). BMI, Body Mass Index; FSH: Follicle-stimulating hormone; LH: Luteinizing hormone; TSH, thyroid-stimulating hormone; FT4, free Thyroxine MII: Metaphase II oocytes.

a Mann-Whitney U test.

b Fisher’s Exact test.

## DISCUSSION

The present study found a statistically significant difference in clinical pregnancy rates in women who underwent embryo transfers with either D5 or D6 blastocysts, which favoured the D5 group. This indicates a weak but significant association between the embryonic culture day and gestational outcome. However, no statistically significant differences were found in miscarriage and live birth rates relative to the frequency of clinical pregnancy. This indicates that once a clinical pregnancy is confirmed, the frequency of live births does not differ between groups with embryos cultured on the 5th or 6th day of blastulation.

Coticchio *et al.* (2023) highlighted that when analysing the absolute frequency of gestational outcomes according to D5 or D6 blastocysts, there was a lower odds ratio for implantation, clinical pregnancy, ongoing pregnancy, and live births, as well as a higher chance of miscarriage and early pregnancy loss in the D6 group (Coticchio *et al*., 2023). These findings are supported by other studies (Park *et al*., 2020) that reported clinical pregnancy rates favour the D5 group, even when evaluating independent factors such as embryo quality (Haas *et al*., 2016; Yerushalmi *et al*., 2021). However, these data remain controversial (Capalbo *et al*., 2014; Jiang *et al*., 2023; Yang *et al*., 2016; Zhang *et al*., 2023).

On the other hand, Capalbo *et al*. (2014) found through logistic regression that morphology and developmental rates were not predictive factors for the implantation potential of euploid embryos in FET cycles, and also noted no differences in implantation rates between D5 and D6 blastocysts. Meanwhile, Andrabi *et al.* (2022) found no statistically significant differences between the D5 and D6 groups in terms of implantation, clinical pregnancy, or miscarriage rates; differences were observed only in live birth rates, which are in contrast to the findings of the present study.

Previous published reports support this finding, showing a considerably lower rate of live births with embryos transferred on D6 than in those transferred on D5 (Chen *et al*., 2023). Similarly, another study conducted in the United States observed no statistically significant differences in implantation rates, pregnancy losses, or pregnancy continuation between D5 and D6 embryos (Whitney *et al*., 2019). These findings contrast with those of the present study, as it was observed that embryos transferred on D5 had lower clinical pregnancy rates, but not lower live birth rates.

Although a systematic review with meta-analysis emphasised suggested that vitrified and warmed embryos on D5 outperform those on D6 in terms of gestational outcomes, such as clinical pregnancy, ongoing pregnancy, and live births (Li *et al*., 2020). We believe that these differences arise from the utilisation of absolute frequency in some studies to assess miscarriage and live birth outcomes. When evaluated in terms of the relative frequency, these data did not differ. The present study highlights the possibility that the poorer gestational outcomes of embryos transferred on D6 may be linked to lower rates of clinical pregnancy. However. once pregnancy occurred, there were no statistically significant differences between miscarriage and live birth rates.

The statistically significant difference in age observed between the participants in the D5 and D6 groups may have interfered with the analysis of these results, even though no participants in this study were aged 45 years or older. However, it is important to emphasize that all patients underwent the same clinical and hormonal evaluation criteria for embryo transfer. Thus, no differences were observed in the other baseline variables that may interfere with reproductive outcomes, indicating a reduction in bias.

Our findings regarding the gestational outcomes of euthyroid women either positive or negative for TPOAb contrast with some reports in the literature, where they did not observe statistically significant differences in clinical pregnancy, miscarriage, or live birth variables between the groups. However, statistically significant differences were observed in LH and TSH levels, with the latter being of little relevance to gestational outcomes when within reference values and assessed individually (d’Assunção *et al*., 2022; Jin *et al*., 2019). Additionally, there was a weak association between higher rates of twin pregnancies in women negative for TPOAb.

This study observed that autoimmune thyroiditis had no effect on gestational outcomes in euthyroid women. Similarly, in a cohort study conducted in Syria, autoimmune thyroiditis positivity was not significantly correlated with gestational outcomes (Hamad *et al*., 2021). Also, a systematic review and meta-analysis presented findings supporting the notion that autoimmune thyroiditis has no effect on gestational outcomes in euthyroid women undergoing IVF (Venables *et al*., 2020). Some authors, although not reporting differences in gestational outcomes, have provided data suggesting that thyroid autoimmunity might affect other variables, such as reducing serum 25-hydroxy vitamin D concentrations, which could result in fewer high-quality embryos (Liu *et al*., 2023).

Dhillon-Smith & Coomarasamy (2020) conducted a systematic review and meta-analysis and argued that there is a link between TPOAb positivity and higher rates of miscarriage and preterm births, factors which we did not observe statistically significant differences in the present study.

Previous studies have reported that higher titres of TPOAb may be related to miscarriage rates, but these findings vary (Hamad *et al*., 2021; Inagaki *et al*., 2020). In this context, we assume that various factors could influence gestational outcomes in TPOAb-positive patients, necessitating further studies that systematically assess TPOAb titres and gestational outcomes.

The present study has several intrinsic limitations owing to its retrospective and single-centre nature that make it challenging to conduct a more comprehensive and longitudinal comparison of outcomes. This raises questions about whether similar results would be observed in different study locations. Therefore, independent verification in other populations is necessary. However, this study is significant because it represents the first Brazilian study to address blastocyst FET on D5 and D6, along with the impact of TPOAb on gestational outcomes.

Other limitations include the absence of individual evaluations of embryo quality and expansiveness as predictors of gestational outcomes. Embryonic morphology seems to have an important role in gestational outcomes, especially in non-biopsied embryos, however, there are reports in the literature that in FET with euploid embryos, the context of the present study, there were no statistically significant differences between the morphological groups (Ji *et al*., 2021). Future studies are needed to investigate the impact of embryonic morphology on gestational outcomes. Nonetheless, we addressed the limitations observed in other studies, such as disproportionality in blastocyst transfer samples (Andrabi *et al*., 2022).

Also, there is a need for studies involving longitudinal follow-up of newborns to assess the potential effect of embryo culture time on neonatal developmental outcomes.

## CONCLUSION

In summary, this study contributes to the literature regarding embryo culture time and clinical outcomes by demonstrating higher rates of clinical pregnancy resulting from the transfer of embryos on D5 than on D6. Our data also indicates no statistically significant differences between the groups in the rates of miscarriage, live births, and prematurity when assessed in terms of relative frequency. Nevertheless, our results do not support the notion that clinical practices should be altered, or that all populations will yield the same results. This emphasises the need for future multicentre studies with larger sample sizes.
